# Prediction of Genetic Groups within *Brettanomyces bruxellensis* through Cell Morphology Using a Deep Learning Tool

**DOI:** 10.3390/jof7080581

**Published:** 2021-07-21

**Authors:** Manon Lebleux, Emmanuel Denimal, Déborah De Oliveira, Ambroise Marin, Nicolas Desroche, Hervé Alexandre, Stéphanie Weidmann, Sandrine Rousseaux

**Affiliations:** 1 Laboratoire VAlMiS-IUVV, AgroSup Dijon, UMR PAM A 02.102, University Bourgogne Franche-Comté, F-21000 Dijon, France; deborah10130@hotmail.fr (D.D.O.); rvalex@u-bourgogne.fr (H.A.); stephanie.weidmann@u-bourgogne.fr (S.W.); sandrine.rousseaux@u-bourgogne.fr (S.R.); 2 AgroSup Dijon, Direction Scientifique, Appui à la Recherche, 26 Boulevard Docteur Petitjean, F-21000 Dijon, France; emmanuel.denimal@agrosupdijon.fr; 3 Plateau D’imagerie DimaCell, Esplanade Erasme, Agrosup Dijon, UMR PAM A 02.102, University Bourgogne Franche-Comté, F-21000 Dijon, France; ambroise.marin@agrosupdijon.fr; 4 Nexidia S.A.S., 15 Rue de Mayence, 21000 Dijon, France; nicolas.desroche@nexidia.fr

**Keywords:** *Brettanomyces bruxellensis*, morphology, genetic groups, deep learning, RAPD-PCR

## Abstract

*Brettanomyces bruxellensis* is described as a wine spoilage yeast with many mainly strain-dependent genetic characteristics, bestowing tolerance against environmental stresses and persistence during the winemaking process. Thus, it is essential to discriminate *B. bruxellensis* isolates at the strain level in order to predict their stress resistance capacities. Few predictive tools are available to reveal intraspecific diversity within *B. bruxellensis* species; also, they require expertise and can be expensive. In this study, a Random Amplified Polymorphic DNA (RAPD) adapted PCR method was used with three different primers to discriminate 74 different *B. bruxellensis* isolates. High correlation between the results of this method using the primer OPA-09 and those of a previous microsatellite analysis was obtained, allowing us to cluster the isolates among four genetic groups more quickly and cheaply than microsatellite analysis. To make analysis even faster, we further investigated the correlation suggested in a previous study between genetic groups and cell polymorphism using the analysis of optical microscopy images via deep learning. A Convolutional Neural Network (CNN) was trained to predict the genetic group of *B. bruxellensis* isolates with 96.6% accuracy. These methods make intraspecific discrimination among *B. bruxellensis* species faster, simpler and less costly. These results open up very promising new perspectives in oenology for the study of microbial ecosystems.

## 1. Introduction

The spoilage yeast *Brettanomyces bruxellensis* presents many strain dependent characteristics, such as volatile phenol production that contributes to the famous “Brett character” [1,2,3], or capacities to withstand many stresses associated with wine related environments (nutritional requirements, resistance to low pH values, capacity to enter in Viable But Not Cultivable state and SO_2_ resistance) [4,5,6,7,8]. In addition to this ability of *B. bruxellensis* to persist in wine [7,9,10,11] and in cellars [12,13], the evolution of oenological practices, such as reducing oenological inputs (like lower SO_2_ doses) and the impact of climate change on the physicochemical characteristics of wines (like higher pH levels), make the control of *B. bruxellensis* more challenging [14,15] and, therefore, leads to serious financial losses for winemakers [16]. Consequently, it is important to develop tools to further discriminate from the species level toward the strain level, and therefore, potentially, to predict spoilage-related phenotypes.

A large number of methods have been developed to discriminate yeasts at the strain level, but few studies have investigated intraspecific diversity among the species *B. bruxellensis*, mostly by distinguishing groups of strains. All of the studies described molecular methods, except one, which used Fourier Transform Infrared Attenuated Total Reflectance (FTIR-ATR) spectroscopy to analyze the molecular composition of cells with mid-infrared radiation for *B. bruxellensis* strain discrimination. Despite being rapid and not requiring DNA extraction, this method cannot be applied routinely due to the need to standardize the protocol and access a database specific to the target microorganism [17].

Besides, molecular methods need a DNA extraction step and some of them used restriction enzymes like the Mitochondrial DNA Restriction Fragment Length Polymorphism (mtDNA RFLP) method [18,19,20] and the Restriction Endonuclease Analysis Pulse Field Gel Electrophoresis (REA-PFGE) method [21]. Then, methods combining the use of restriction enzymes and Polymerase Chain Reaction (PCR) allowed for the increase in discriminating power of the analysis, such as Amplified Fragment-Length Polymorphism (AFLP) amplification [22,23] and the Sau-PCR method [21,24,25]. However, these methods that use restriction enzymes are quite laborious and can be difficult to interpret due to artifacts in electrophoresis [18].

Then, the methods that followed were based only on PCR. One of them was the Intron Splice Site PCR (ISS-PCR) method [18,26,27]. Recently, microsatellite analysis was applied for intraspecific discrimination among *B. bruxellensis* species [21,28]. This technique became a highly discriminating, robust and reproducible method adapted to the study of large populations, discriminating strains in different genetic groups and providing information in particular about their ploidy state and SO_2_ resistance [5,12,29,30]. Thus, the determination of the genetic group of a given isolate could allow for predicting the risk of SO_2_ resistance and, thus, help winemakers to adapt their antimicrobial techniques [5]. In addition, a diagnostic tool, TYPEBrett (ISVV-Microflora patent PCT/FR2016/052701) based on the microsatellite technique was developed and patented. Another PCR-based technique was the Random Amplified Polymorphism DNA PCR (RAPD-PCR) method. This method was most widely used to study intraspecific diversity among *B. bruxellensis* species, even if it could sometimes be combined with other methods to improve discrimination [19,21,31,32,33,34,35,36,37]. The first use of RAPD-PCR provided poor discrimination of wine isolates from reference strains [31], but the method was improved by combining the individual profiles of each primer to assign composite profiles to each strain, making it more discriminating [19]. However, RAPD-PCR has sometimes been described as poorly reproducible and less efficient than other methods, but these problems could be minimized by standardizing the protocol [21,38]. Therefore, the RAPD-PCR method presents many advantages such as low cost, ease of use and analysis [39] to reveal intraspecific diversity within *B. bruxellensis* species, even if molecular methods require advanced skills and specialized equipment.

Another direction could be explored in order to further simplify discrimination. A previous work described different cell morphologies depending on the strain, and suggested a link between the variability in yeast cell shapes and the genetic group to which they belong [11]. To validate this suggested link, extensive analyses through larger microscopic observations with a consequent number of isolates should be conducted. In this context, the use of tools such as those provided by deep learning methods appears obvious. Deep learning algorithms have been developed to classify images into defined categories by exploiting available features they contain [40,41]. In recent years, such algorithms have been applied to images in various biological fields and have transformed the analysis and interpretation of imaging data. For example, deep learning tools have been used to discriminate foodborne pathogens [42], to aid in sickle cell anemia diagnosis [43] and to predict mechanisms of drug resistance of cancer cells [44]. The aim of the present work is to provide rapid, easy-to-use and inexpensive tools for routine intraspecific discrimination. First, a RAPD-PCR protocol was adapted to study intraspecific diversity among 74 isolates belonging to four genetic groups, and provides a baseline for classifying these isolates using a simple and accessible molecular technique. Simultaneously, a pre-trained Convolutional Neural Network (CNN) was fitted to predict the genetic group of isolates tested based on differences in cell morphology.

## 2. Materials and Methods

### 2.1. Yeast Isolates and Strains

This study included a total of 74 isolates previously identified as *B. bruxellensis* and discriminated in four different genetic groups (GG1, GG2, GG3 and GG4) by microsatellite analysis (Table 1): (i) 10 strains of *B. bruxellensis* from collections (AWRI1499, CDR3, CDR9, CDR11, CDR12, CDR217, CDR219, LO2E2, LO417, LO6/036) [29], and (ii) 64 isolates obtained from enological materials and/or wine from wineries (named from 1 to 64) [11]. The yeasts were stored at −80 °C in YPD liquid medium (0.5% *w*/*v* yeast extract (Biokar, Beauvais, France), 1% *w*/*v* bactopeptone (Biokar), 2% *w*/*v* D-glucose (Prolabo, Fontenay-sous-Bois, France) and 0.02% *w*/*v* chloramphenicol (Sigma, St Louis, MI, USA), containing 20% *v*/*v* glycerol.

### 2.2. Genetic Analysis

#### 2.2.1. DNA Extraction

Using stock-cultures stored at −80 °C, cultures were prepared in YPD medium at 28 °C. After growth until the stationary phase, cells were harvested by centrifugation (9150× *g* for 4 min at 4 °C), washed with 1 mL of sterile water and then re-suspended in 200 µL of extraction buffer (SDS 1% *w*/*v*, Triton × 100 2% *w*/*v*, NaCl 100 mM, Tris 10 mM, EDTA 1 mM, pH = 8), with 0.3 g glass beads (0.5 mm in diameter; Scientific Industries) and 60 µL phenol:chloroform:isoamyl alcohol 25:24:1 (Sigma). The cells were broken for 3 × 45 s at 6500 rpm with a 60-s interval using a Precellys 24-Dual Homogenizer (Bertin Technologies, Montigny-le-Bretonneux, France). After the addition of 200 µL of Tris EDTA buffer (Tris 10 mM, EDTA 1 mM, pH 8), the cultures were centrifuged at 18,200× *g* for 10 min at 4 °C. The aqueous phase was collected and the DNA was precipitated with 1 mL of 100% ethanol and then centrifuged at 18,200× *g* for 10 min at 20 °C. The pellet was washed with 70% ethanol and centrifuged at 18,200× *g* for 5 min at 20 °C. Then, the pellet was dried and resuspended in sterile water. Finally, the samples were stored at −20 °C. The concentration and purity of DNA was measured by Infinite M200 Pro NanoQuant (TECAN, Lyon, France).

#### 2.2.2. RAPD-PCR

RAPD profiles were obtained using 3 primers, OPA-02 (5′-TGC CGA GCT G-3′), OPA-03 (5′-AGT CAG CCA G-3′) and OPA-09 (5′-GGG TAA CGC C-3′) (Eurogentec, Seraing, Belgium), as previously described [19,21,31,33]. PCR reactions were performed in 40 µL reactions containing 50 ng of DNA, Go Taq Buffer 1.25×, 1.875 mM MgCl_2_, 2 µM of primer (1 primer per PCR reaction), 0.25 mM of each dNTP and 2.5 U of Go Taq polymerase (Go Taq^®^ Flexi DNA Polymerase, Promega, Madison, WI, USA). The amplification reactions were carried out in a thermocycler BioRAD T100™ (BioRAD, Marnes-la-Coquette, France) under the following conditions: initial denaturation at 95 °C for 5 min, followed by 40 cycles of 94 °C for 1 min, 36 °C for 1 min, 72 °C for 2 min and a final extension at 72 °C for 10 min [19,33]. The reaction products were analyzed by capillary electrophoresis using MultiNA (MCE-202/MultiNA, Shimadzu Biotech, Marne la Vallée, France) and the DNA-2500 Reagent Kit (Shimadzu Corporation, Marne la Vallée, France) with pGEM® DNA Markers (Promega, Madison, USA) as ladder.

### 2.3. Cell Polymorphism Analysis

#### 2.3.1. Cultures

Isolates were grown on YPD plates (YPD broth with 2% *w*/*v* agar) at 28 °C from the stock at −80 °C. From each YPD plate, one isolated colony was inoculated in 15 mL of YPD and the liquid cultures were incubated at 28 °C for 72 h.

#### 2.3.2. Microscopic Observations

For each culture, 6 µL was placed between the slide and coverslip and optical microscopic transmitted light observations were performed. Images were scanned at 1280 × 960 pixels, 8-bit gray scale, at ×40 magnification and 38% brightness using the EVOS^®^ FL Imaging System (Invitrogen, Bothell, WA, USA).

Two data sets were built.

Dataset1 was composed of 10 images per culture and 3 independent cultures for each of the 74 isolates. Six images of isolate 30 and one of isolate 56 were rejected due to artifacts. The distribution of images among genetic groups was the following: 354 images for GG1, 210 for GG2, 1499 for GG3 and 150 for GG4.

Dataset2 consisted of 233 images, with 46 images for GG1 (10 images for 1 culture of isolate 14; 17 images for 2 cultures of isolate 49; 19 images for 2 cultures strain LO2E2), 63 images for GG2 (10 images for 1 culture of isolate 2; 18 images for 2 cultures of isolate 11; 18 images for 2 cultures of isolate 17; 17 images for 2 cultures of isolate 20), 66 images for GG3 (10 images for 1 culture of isolate 7; 19 images for 2 cultures of isolate 9; 18 images for 2 cultures of isolate 32; 19 images for 2 cultures of isolate 60) and 58 images for GG4 (10 images for 1 culture of isolate 63; 16 images for 2 cultures of strain CDR219; 16 images for 2 cultures of strain LO417; 16 images for 2 cultures of strain AWRI1499).

#### 2.3.3. Cell Shape Determination

From dataset1, one representative (without artifact, similar cell density) image per biological replicate was selected, amounting to 3 images per isolate. A total of 100 cells were pseudo-randomly selected (excluding buds) from these 3 images. Then, ImageJ software (1.52i) was used to measure cell morphology characteristics as described in a previous study [11]. Briefly, the length to width (*l*/*w*) ratio and the cell area were determined from the measurement of 100 single cells per isolate, amounting to 7400 cells.

#### 2.3.4. Deep Learning

The pre-trained Convolutional Neural Network GoogleNet was chosen. This CNN was trained with over 1 million images classified into 1000 object categories. This network thus learned rich feature representations for a wide range of images. It had to be adapted by transfer learning to specialize it for the discrimination of genetic groups. Hence, the end of the CNN was customized to output 4 categories, corresponding to the 4 genetic groups instead of the 1000 initial object categories. Then, the learning rate weight of the last layers was set to 5 to accentuate the learning rate. On the contrary, the learning rate weight of the 10 first layers was frozen in order to keep the advantage of the previous training.

**Figure 1:**
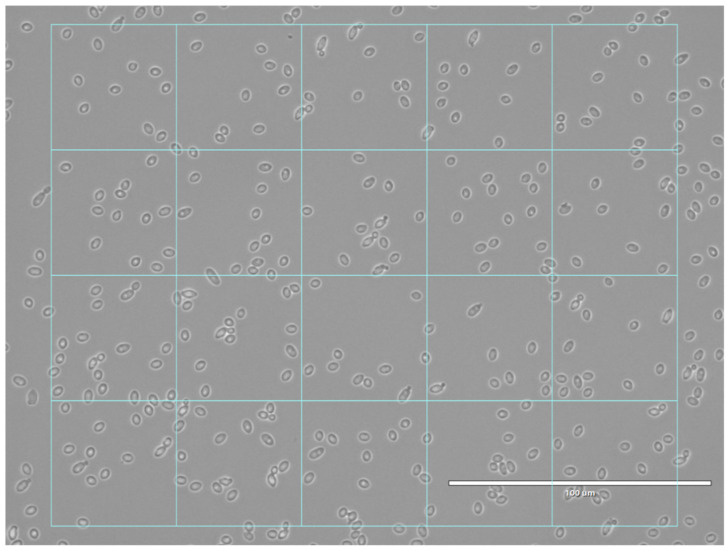
Typical microscopic image divided into thumbnails.

Firstly, dataset1 was used to train the CNN. To fit the input requirement of the network and increase the number of learning cases, the microscopic images were divided into subpictures (Figure 1). To do this, each image from dataset1 was divided into multiple thumbnail images of 224 × 224 pixels, corresponding to the input size of the pre-trained CNN. Several thousand thumbnails were generated in this way. To balance the sets for each genetic group, the same number (3000) of randomly selected thumbnails were used, corresponding to the number of elements in the smallest genetic group (GG4). This 4 × 3000 thumbnail dataset was then randomly divided into two subsets: (i) 75% used for CNN training (training dataset), and (ii) 25% used for training performance validation (validation dataset) and the calculation of the confusion matrix.

Due to the low number of images available, data augmentation was performed to increase the amount of training data. The augmentation process was achieved by applying several random transformations from original images consisting of rotations (−45°, +45°), horizontal and vertical mirrors and *x*-, *y*-axis translations (−30 pixels, +30 pixels). The training was performed on 64 epochs using a Nvidia GTX1080 GPU.

Secondly, dataset2 was used to test the accuracy of the trained model. For each of the 233 images to be classified, the same thumbnailing process as for the dataset1 was applied. Then, each thumbnail of the image to be labeled was presented to the CNN, which associated it with one genetic group. The genetic group that was most frequently assigned among the thumbnails was then assigned to the image.

## 3. Results and Discussion

### 3.1. Intraspecific Discrimination of the B. bruxellensis Isolates into Genetic Groups

**Figure 2:**
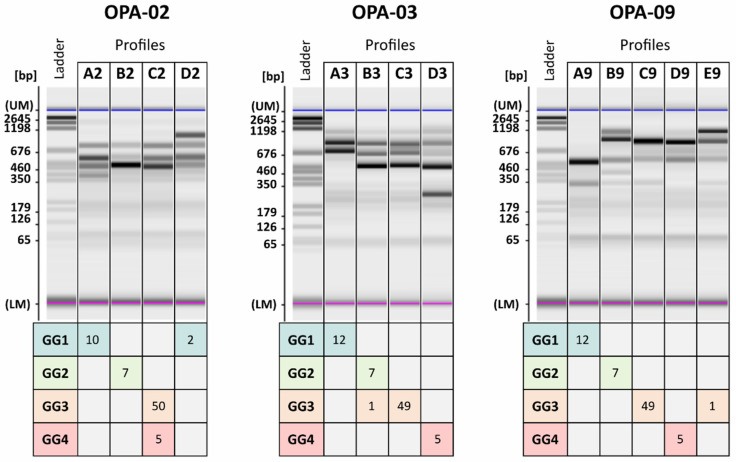
Random Amplified Polymorphic DNA PCR (RAPD-PCR) profiles obtained with each primer, OPA-02, OPA-03 and OPA-09, for 74 isolates of *Brettanomyces bruxellensis*. Profiles are indicated by different letters for each primer. Genetic groups are indicated on the left and by different color. At the intersection of a row (genetic group) and a column (RAPD-PCR profile) is the number of isolates corresponding to a given combination of a genetic group and a RAPD-PCR profile. Ladder: pGEM® DNA Markers.

RAPD-PCR was adapted here to assess its discriminating power and provide an accessible protocol to determine the genetic group of *B. bruxellensis* isolates. RAPD-PCR assay was performed on 74 *B. bruxellensis* isolates with 3 different primers among those most used in the literature: OPA-02, OPA-03 and OPA-09 [19,21,31,33]. The analysis led to obtaining 4 profiles for OPA-02 (A2 to D2) and OPA-03 (A3 to D3), and 5 profiles for OPA-09 (A9 to F9) (Figure 2), making OPA-09 the most discriminating primer compared to the other two primers, in accordance with previous studies [19,31]. The reproducibility of the RAPD-PCR method adapted in this study was checked (as Supplementary Data, Figure S1).

The ability to predict a *B. bruxellensis* isolate’s adaptability to given environmental factors, such as SO_2_ resistance, was demonstrated by determining a genetic group of isolates using microsatellite genotyping [5]. In this work, we investigated whether the RAPD-PCR adapted method could assign genetic groups in the same way as microsatellite genotyping. This was done with the aim of proposing a routine method to potentially predict SO_2_ resistance. Therefore, the intraspecific diversity obtained by RAPD-PCR was compared to the discrimination in genetic groups by microsatellite analysis (Figure 2). Whatever the primer used, high correlation was obtained between genetic groups and RAPD-PCR profiles. Nevertheless, primer OPA-02 did not discriminate between GG3 and GG4, which shared the same profile C2. Similarly for primer OPA-03, GG2 and GG3 shared the same profile B3. The best correlation was obtained with primer OPA-09 since each profile corresponded to a unique GG: profile A9 to GG1, B9 to GG2 and D9 to GG4. GG3 was divided into 2 profiles, C9 and E9. Thus, RAPD-PCR was suitable for discriminating the 74 isolates of *B. bruxellensis* in the same distribution as microsatellite analysis within genetic groups described [11,29]. This RAPD-PCR protocol could be applied to provide a simple and rapid molecular means of determining the genetic group of a *B. bruxellensis* isolate, and thus, a prediction of its phenotype since the identification of the genetic group of a given isolate provides information about its SO_2_ resistance [5].

### 3.2. From Cell Polymorphism to Genetic Groups

#### 3.2.1. Qualitative and Quantitative Description of Cell Morphology

In the literature, microscopic observations of *B. bruxellensis* strains show a high level of cell polymorphism. Up to now, the majority of the studies reporting polymorphism in *B. bruxellensis* have focused on its ability to form filaments, described as a survival mechanism [45]. Thus, the description of polymorphism as strain dependent mainly focused on filaments [46,47,48,49] at the expense of yeast cells, which display highly diverse morphology and are not sufficiently exploited. Indeed, correlating the diversity of yeast cell morphology to an intraspecific genetic diversity is difficult due to the limited data available, making it difficult to compare the results of the different studies (few or no microscopic observations available, heterogeneous images, different culture conditions). Nevertheless, a previous study suggested that the variability of the yeast cell shapes may be linked to intraspecific diversity within *B. bruxellensis* species. Indeed, microscopic observations of 12 strains of *B. bruxellensis* revealed different cell shapes depending on the strain, and that this shape variability was linked to the genetic group of the strains [11]. Thus, this phenotypic characteristic could be a tool for discriminating isolates.

**Figure 3:**
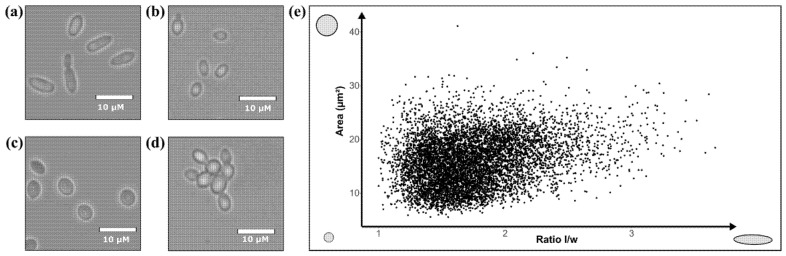
Polymorphism of yeast cells among 74 *Brettanomyces bruxellensis* isolates: (**a**) elongated cells, (**b**) small cells, (**c**) round cells and (**d**) presence of multicellular structure. (**e**) Distribution of 7400 cells of *B. bruxellensis* (100 cells per strain) according to length to width (*l*/*w*) ratio and area measurements.

In this work, 74 isolates of *B. bruxellensis* were observed by transmitted light optical microscopy, highlighting different cell features. First, several distinctive cell shapes appeared according to the strain: elongated cells (Figure 3a), small cells (Figure 3b) and round cells (Figure 3c). No influence of growth stage, culture conditions and lifestyle on cell morphology was noticed (data not shown), confirming previous studies [11,46]. These various shapes were consistent with previous studies, each of which described a range of cell shapes such as round, elongated, ovoid, carrot-shaped and ellipsoidal [11,33,46,47,48,50,51]. In addition, strains belonging to GG4 presented the characteristic of forming multicellular structures (aggregation of independent cells or non-separation of daughter cells) (Figure 3d), which cannot be described through individual cell shape. Strains belonging to the other 3 genetic groups only rarely displayed these multicellular structures. This feature has rarely been described in *B. bruxellensis* species [46,51].

Then, to further investigate the link between yeast cell morphology and genetic groups, it was necessary to compare a large number of cells and rely on quantitative data. Thus, the qualitative feature “shape” was transposed into a quantitative variable by plotting the cell area versus the *l*/*w* ratio (Figure 3e). The shapes of 100 cells for each of the 74 isolates, totaling 7400 cells, from dataset1 were assessed: the higher the ratio, the more elongated the cells and the higher the area value, the bigger the cells. The wide distribution of the points highlighted highly diverse cell morphology in the species *B. bruxellensis*.

**Figure 4:**
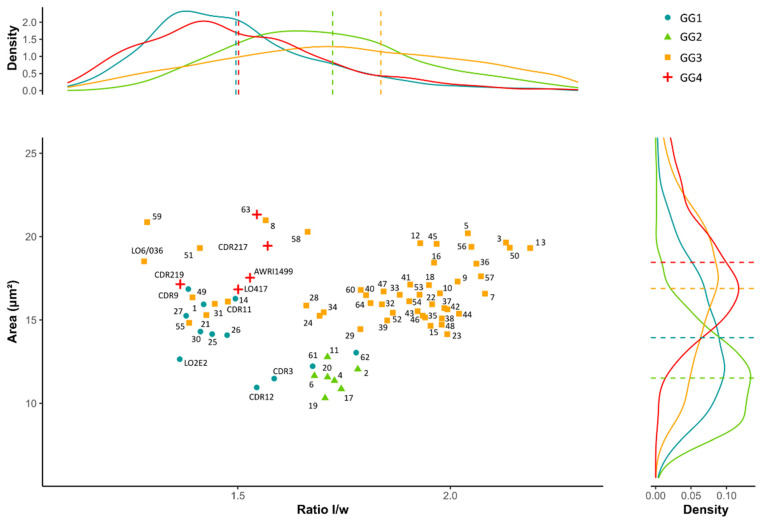
Distribution of 74 isolates of *Brettanomyces bruxellensis* according to average length to width (*l*/*w*) ratio and average area measurements. For each genetic group, the density curves for the *l*/*w* ratio (on the **top**) and for the area (on the **right**) were assigned with the mean (dashed line). Each genetic group is represented by a different color: GG1 (blue), GG2 (green), GG3 (orange) and GG4 (red).

To investigate this distribution further, density curves were plotted according to the area on the right and to the *l*/*w* ratio on the top, to show the distribution of the cells inside each genetic group (Figure 4). GG1 and GG4 presented a low average *l*/*w* ratio (1.50), corresponding to rounder cells. However, GG1 presented a lower average area (13.9 µm^2^) than GG4 (18.5 µm^2^). GG2 and GG3 presented higher average *l*/*w* ratios (1.72 and 1.84, respectively), corresponding to more elongated cells. Moreover, these groups could be discriminated by their average area, 11.5 µm^2^ for GG2 corresponding to small cells, compared to 16.9 µm^2^ for GG3. Differences appeared between genetic groups, but considering the spread of the curves, overlaps suggested similarities in cell morphology between genetic groups. In particular, the curves corresponding to GG3 had a large amplitude. As such, to refine the discrimination according to cell morphology, it would be relevant to explore it at the isolate level. Therefore, the average *l*/*w* ratio and the average area of 100 cells were determined for each isolate. The 2D spatial representation of these averages specified the clusters according to genetic groups (Figure 4). GG2 was clearly separated from GG3 and GG4, while close to some isolates of GG1. The large majority of GG3, corresponding to elongated cells, was also clearly separated. However, it should be noted that some isolates belonging to GG3 had a lower *l*/*w* ratio and were mixed with several GG1 and GG4 isolates. These latter isolates, from GG4, were also grouped together. Despite some minor confusions between the different groups, it is clear that, in the culture conditions of this study, the polymorphism of the cells was related to genetic groups.

Nevertheless, this manual treatment of the images quickly limited the analysis since it was time consuming, quite subjective and it took account of only two cell shape attributes, leaving out the distinctive feature of GG4 to form multicellular structures although it could be a key point for genetic group discrimination. Thus, in order to analyze all cell and population features available through microscopic images rapidly and reproducibly, a more robust method such as deep learning could be applied. Indeed, algorithms for deep learning, such as convolutional neural networks, allow image classifying into defined categories by exploitation of all the features available in raw images (even the subtlest ones) and automatic learning [40,41].

#### 3.2.2. Use of Deep Leaning to Predict the Genetic Group of *B. bruxellensis* Isolates

For the present study, the use of a CNN allows us to change the focus from studying single cells to that of the general aspect of the population. Indeed, although the different classes share common shapes, the algorithm ought to separate them according to their differences, and thus, the majority shapes and behaviors.

To exploit intra- and inter-genetic group variability more efficiently, the CNN GoogleNet was used and trained to classify microscopic images in the 4 genetic groups. The detailed performance analysis is shown in Table 2. The model successfully predicted the genetic group of the image 90.8% of the time, with an F1-score of 0.9076. The model was 91.05% reliable when assigning a genetic group to an image. These results indicated that the model was able to predict the genetic group of an isolate from a simple microscopic observation more than 9 times out of 10.

**Figure 5:**
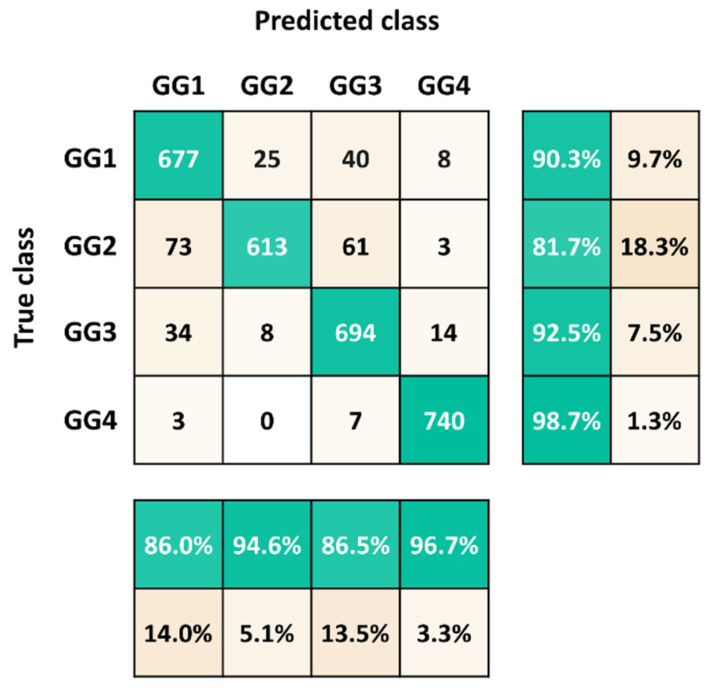
Confusion matrix for the classification of 4 genetic groups from dataset1.

Results of classification for dataset validation (from dataset1) are presented in the confusion matrix (Figure 5). The diagonal represented the correctly predicted number of each observation. The precision (bottom) and the sensitivity (right column) were calculated for each class. Each genetic group was predicted at 90.3%, 81.7%, 92.5% and 98.7% for GG1, GG2, GG3 and GG4, respectively. Moreover, the model reached a high prediction reliability of 86.0%, 94.6%, 86.5% and 96.7% for GG1, GG2, GG3 and GG4, respectively. The minor confusions between genetic groups could be explained by the characteristics they share, like GG1 and some GG3 isolates clustered together due to a low *l*/*w* ratio in Figure 4. In addition, GG4 was the best predicted class probably due to the behavior characteristic of this genetic group, which tends to form multicellular structures and is, therefore, easy to discriminate (Figure 3d). The classification results indicated the model clearly learnt to classify images based on cell morphological and population features.

To test the “real-world” performance of the model, classification was performed on dataset2. With these microscopic images, the model achieved an accuracy of 96.6%, confirming its outstanding predictive power. Thus, this deep learning method stands as a prime method for the study of microbial intraspecific discrimination. Indeed, from a simple microscopic observation of a culture of *B. bruxellensis*, it is possible to predict the genetic group of the isolate studied with fairly high confidence. The prediction of the CNN might achieve greater accuracy by including more strains, since the larger and more representative the training database, the better the performances of the CNN. The CNN trained in this study provides a very fast initial result, which can be used as is. Nevertheless, if a higher level of confidence is required, it can be achieved with the RAPD-PCR protocol. This allows confirmation at the genetic level of the pre-determination in silico of the genetic group. The complementarity of both methods makes intraspecific discrimination within *B. bruxellensis* species efficient, rapid, simple and inexpensive.

#### 3.2.3. Does the Link between Genetic Groups and Cell Morphologies Predict any Specific Functions?

The existence of a link between cell polymorphism and genetic groups in the *B. bruxellensis* species indicates that shape variation is probably linked to clusters of genes encoding for various functions, which could be related to adaptation to environmental factors, for example.

In a study investigating the phenotype of a nearly complete collection of gene-deletion mutants of *Saccharomyces cerevisiae* [52], 673 strains exhibited slight to strong morphological alterations compared to a wild-type strain, allowing the identification of genes involved in specifying cell shape and size. In particular, clumped and elongated strains were enriched for mutations in genes for cell growth, cell division and DNA synthesis, whereas round strains were enriched for mutations in protein synthesis genes. Therefore, in the *B. bruxellensis* species, since the genetic groups gather strains with the same morphological typology, it is likely that strains from the same genetic group share similar mutation types.

In addition, microsatellite analysis revealed that strains of *B. bruxellensis* were structured in genetic groups according to ploidy level [29]. Thus, cell morphology may be related to ploidy level, especially as another morphological characteristic, filamentation, has been shown to be related to the ploidy of yeasts. Indeed, in *S. cerevisiae*, different triggers for pseudohyphae formation have been reported for diploid (nitrogen limitation) and haploid cells (glucose deprivation) [46,53,54,55].

Besides the shapes, one of the genetic groups was distinguishable by a multicellular phenotype. Such phenotypes, like flocs resulting from aggregation or clumps formed by incomplete separation, have been described in *S. cerevisiae* as protection mechanisms against environmental stresses [56,57,58,59]. In *B. bruxellensis*, it turns out that GG4, which presented this feature, is mainly composed of SO_2_ resistant/tolerant strains [5]. Thus, these multicellular structures may be involved in the SO_2_ resistance of the yeast.

## 4. Conclusions

In this study, the CNN GoogleNet was trained to provide rapid and highly reliable screening of genetic groups of *B. bruxellensis* isolates, since it was able to correctly predict the genetic group of an isolate from a simple microscopic observation with 96.6% accuracy. This CNN provides a rapid, simple and inexpensive tool for routine intraspecific discrimination in *B. bruxellensis* species. Pre-determination in silico of the genetic group of an isolate can be confirmed at the genetic level by the RAPD-PCR protocol, since the use of primer OPA-09 successfully discriminated *B. bruxellensis* isolates in the same distribution among genetic groups as microsatellite analysis, in a more accessible and less expensive manner.

The present study opens new perspectives for the use of deep-learning methods in oenology to provide powerful, robust and timesaving analyses. The polymorphism of yeast cells among *B. bruxellensis* species clearly appears to be related to the genetic group of isolates. Therefore, assessing cell morphology could allow us to predict the genetic group of isolates, itself linked to their SO_2_ resistance. The same intraspecific discrimination study could be considered for other yeast species using a CNN. Furthermore, discrimination could be extended at the interspecies level for the identification of unknown microorganisms. CNN could be used to pre-classify species of yeast or species of bacteria from routine microscopic observations performed after isolation to optimize subsequent molecular analyses. Moreover, this tool could be useful for implantation control and population monitoring during winemaking, providing real-time results simply by microscopic observation.

## Figures and Tables

**Table 1:** List of isolates and strains used in this study.

Isolate/Strain of Reference	Genetic Group (GG)
1, 14, 25, 26, 27, 30, 49, 61, 62, LO2E2, CDR3, CDR12	GG1
2, 4, 6, 11, 17, 19, 20	GG2
3, 5, 7, 8, 9, 10, 12, 13, 15, 16, 18, 21, 22, 23, 24, 28, 29, 31, 32, 33, 34, 35, 36, 37, 38, 39, 40, 41, 42, 43, 44, 45, 46, 47, 48, 50, 51, 52, 53, 54, 55, 56, 57, 58, 59, 60, 64, LO6/036, CDR9, CDR11	GG3
63, CDR217, CDR219, LO417, AWRI1499	GG4

AWRI: Australian Wine Research Institute, Australia; CDR: Côtes du Rhône, Inter Rhône, France.

**Table 2:** Performance of the Convolutional Neural Network (CNN) GoogleNet trained and validated with dataset1 to classify genetic groups from microscopic images.

Accuracy	Error	Sensitivity	Specificity	Precision	FalsePositive Rate	F1-Score
0.9080	0.0920	0.9080	0.9693	0.9105	0.0307	0.9076

## Supplementary Materials

The following are available online at https://www.mdpi.com/article/10.3390/jof7080581/s1, Figure S1: Reproducibility of Random Amplified Polymorphic DNA PCR (RAPD-PCR) adapted method. For each primer (OPA-02, OPA-03 and OPA-09), the profiles of 3 independent repetitions are provided for 3 different isolates. Ladder: pGEM® DNA Markers.
