# Supplementary material for: Prediction of Genetic Groups within Brettanomyces bruxellensis through Cell Morphology Using a Deep Learning Tool
Source: J Fungi (Basel). 2021 Jul 21;7(8):581. doi: 10.3390/jof7080581 (PMC8396822; doi:10.3390/jof7080581)
Supplement: Supplementary file 1 [file jof-07-00581-s001.zip › jof-1296666-supplementary.pdf]

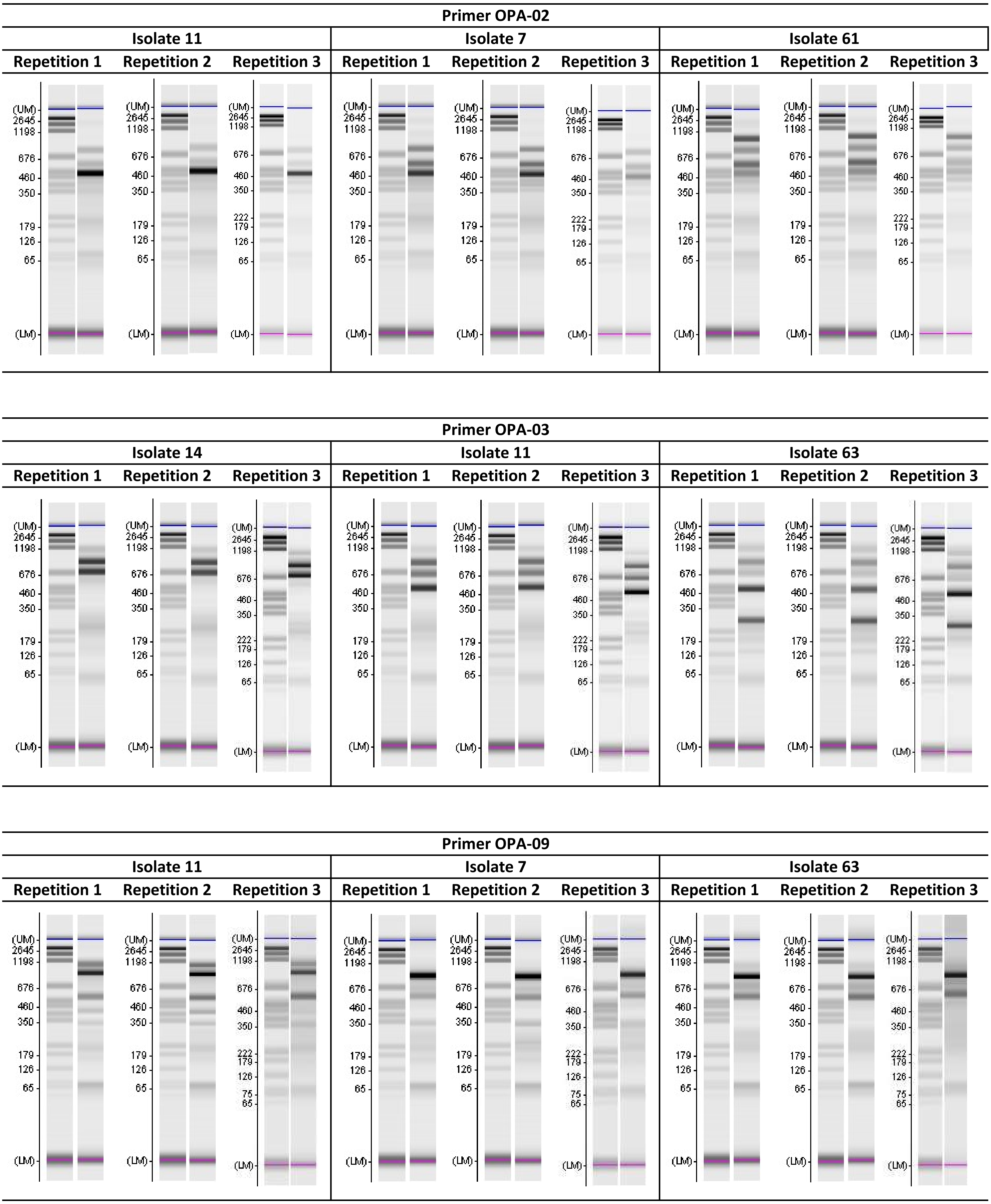

Figure S1: Reproducibility of Random Amplified Polymorphic DNA PCR (RAPD-PCR) adapted method. For each primer (OPA-02, OPA-03 and OPA-09), the profiles of 3 independent repetitions are provided for 3 different isolates. Ladder: pGEM® DNA Markers.
